# Modulation of Androgen Receptor Signaling in Hormonal Therapy-Resistant Prostate Cancer Cell Lines

**DOI:** 10.1371/journal.pone.0023144

**Published:** 2011-08-04

**Authors:** Rute B. Marques, Natasja F. Dits, Sigrun Erkens-Schulze, Wilfred F. J. van IJcken, Wytske M. van Weerden, Guido Jenster

**Affiliations:** 1 Department of Urology, Josephine Nefkens Institute, Rotterdam, The Netherlands; 2 Erasmus Center for Biomics, Erasmus Medical Center, Rotterdam, The Netherlands; Institute of Genetics and Molecular and Cellular Biology, France

## Abstract

**Background:**

Prostate epithelial cells depend on androgens for survival and function. In (early) prostate cancer (PCa) androgens also regulate tumor growth, which is exploited by hormonal therapies in metastatic disease. The aim of the present study was to characterize the androgen receptor (AR) response in hormonal therapy-resistant PC346 cells and identify potential disease markers.

**Methodology/Principal Findings:**

Human 19K oligoarrays were used to establish the androgen-regulated expression profile of androgen-responsive PC346C cells and its derivative therapy-resistant sublines: PC346DCC (vestigial AR levels), PC346Flu1 (AR overexpression) and PC346Flu2 (T877A AR mutation). In total, 107 transcripts were differentially-expressed in PC346C and derivatives after R1881 or hydroxyflutamide stimulations. The AR-regulated expression profiles reflected the AR modifications of respective therapy-resistant sublines: AR overexpression resulted in stronger and broader transcriptional response to R1881 stimulation, AR down-regulation correlated with deficient response of AR-target genes and the T877A mutation resulted in transcriptional response to both R1881 and hydroxyflutamide. This AR-target signature was linked to multiple publicly available cell line and tumor derived PCa databases, revealing that distinct functional clusters were differentially modulated during PCa progression. Differentiation and secretory functions were up-regulated in primary PCa but repressed in metastasis, whereas proliferation, cytoskeletal remodeling and adhesion were overexpressed in metastasis. Finally, the androgen-regulated genes ENDOD1, MCCC2 and ACSL3 were selected as potential disease markers for RT-PCR quantification in a distinct set of human prostate specimens. ENDOD1 and ACSL3 showed down-regulation in high-grade and metastatic PCa, while MCCC2 was overexpressed in low-grade PCa.

**Conclusions/Significance:**

AR modifications altered the transcriptional response to (anti)androgens in therapy-resistant cells. Furthermore, selective down-regulation of genes involved in differentiation and up-regulation of genes promoting proliferation and invasion suggest a disturbed balance between the growth and differentiation functions of the AR pathway during PCa progression. These findings may have implications in the current treatment and development of novel therapeutical approaches for metastatic PCa.

## Introduction

Prostate cancer is the most frequently diagnosed non-cutaneous malignancy in men and the second leading cause of cancer deaths in the western countries [1]. Prostate cancer is a highly heterogeneous condition, exhibiting a wide range of biological and clinical manifestations. While some patients develop an asymptomatic disease course and rather die with the cancer than from the cancer, others present with a more aggressive and/or more advanced disease at the time of diagnosis [2]. When the tumor is confined to the prostate, it can be efficiently treated by radical surgery and/or radiation therapy, but once the tumor has disseminated, systemic therapy is required. Since prostate cancer cells require androgens for their survival and growth, the golden standard for the treatment of evasive prostate tumors is androgen ablation through chemical or surgical castration, which may be combined with the administration of androgen receptor (AR) antagonists [2]. Most patients will benefit from this hormonal therapy, the tumors may shrink and the symptoms ameliorate. However, eventually all cancers will become resistant and recur as therapy-refractory or castration-resistant disease, for which at the moment no curative treatment exists [3], [4]. The AR pathway is very versatile, being involved in many biological processes including cellular proliferation, regulation of apoptosis and differentiation [5]. The balance between these different functions dictates the homeostasis of the prostatic gland. A pertinent question is how prostate cancer cells that are initially dependent on androgens can resume growth in an androgen-deprived environment. One possibility is that prostate cancer cells achieve this by adapting their AR pathway to the low androgen/high antiandrogen levels, for example by mutation, amplification or truncation to a constitutively active AR, deregulation of AR cofactors and/or intratumoral androgen production [6], [7]. On the other hand, cancer cells may activate alternative growth pathways, while shutting down tumor suppressors and apoptotic signals [6], [7].

In the present study, we focused on the role of the AR pathway in prostate cancer progression. The expression pattern of androgen-regulated genes in androgen-responsive and castration-resistant cell lines was established, with the goal to: (i) determine whether the AR pathway is still functionally active in the hormonal therapy-resistant PC346 cells; (ii) identify the mechanism(s) by which the AR pathway may be adjusted to the low androgen/high antiandrogen levels; (iii) identify androgen-regulated genes that could potentially be used in the diagnosis/prognosis of prostate cancer or as a therapeutic target. For this purpose, we used microarray technology to characterize the transcriptional program activated by the synthetic androgen R1881 and the antiandrogen hydroxyflutamide. As model system we used the PC346 cell lines (Table 1): the androgen-responsive PC346C parental cell line and its therapy-resistant derivative sublines PC346DCC, PC346Flu1 and PC346Flu2 [8]. These castration-resistant sublines reproduce common AR modifications observed in therapy-resistant disease: AR down-regulation (PC346DCC), AR mutation (PC346Flu2) and AR overexpression (PC346Flu1), making it a unique and valuable model for this study.

**Table 1:** Characteristics of the PC346 cell line panel: AR status and hormone response.

	PC346C	PC346DCC	PC346Flu1	PC346Flu2
AR status	wt AR	AR low	AR high	T877A AR
PSA expression	+ +	-	+ +	+ +
Growth on steroid-stripped medium	-/+	+ +	+ +	+
Growth with 0.1 nM R1881	+ +	+ +	+	+ +
Growth with 1 µM OH-flutamide	-/+	+ +	+ +	+ +

The detailed characterization of the PC346 cell lines was the aim of a previous manuscript [8].

## Methods

### Ethics Statement

Normal and tumor samples from patients were obtained from the frozen tissue bank of the Erasmus Medical Center (Rotterdam, the Netherlands). The specimens were collected between 1984 and 2001. The experimental protocols were approved by the Erasmus MC Medical Ethics Committee according to the Medical Research Involving Human Subjects Act.

### Reagents and cell lines

The basic culture medium used in the maintenance of PC346 cell lines consisted of DMEM-F12 medium (Cambrex BioWhitaker, Belgium) supplemented with 2% fetal calf serum (FCS; PAN Biotech GmbH, Aidenbach, Germany), 1% insulin-transferrin-selenium (Gibco BRL), 0.01% bovine serum albumin (Boehringer Mannheim, Germany), 10 ng/ml epidermal growth factor (Sigma-Aldrich), penicillin/streptomycin antibiotics (100 U/ml penicillin, 100 mg/ml streptomycin; BioWhitaker, Belgium); plus the following additions: 100 ng/ml fibronectin (Harbor Bio-Products, Tebu-bio, The Netherlands), 20 mg/ml fetuine (ICN Biomedicals, The Netherlands), 50 ng/ml choleratoxin, 0.1 mM phosphoethanolamine, 0.6 ng/ml triiodothyronine and 500 ng/ml dexametason (all from Sigma). PC346C cells were maintained in culture in the complete medium mentioned above, supplemented with 0.1 nM 17-methyltrienolone (R1881; NEN, Boston MA, USA). PC346DCC selection medium was supplemented as described above, but depleted from androgens by using dextran-coated charcoal (DCC) treated FCS. PC346Flu1 and PC346Flu2 culture medium was also androgen depleted by using 2% DCC-FCS, and supplemented with 1 µM of hydroxyflutamide (OH-flutamide, Schering-Plough Research Institute, New Jersey, USA). For the hormone stimulations, a simplified version of the culture medium was used, containing 2% DCC- FCS without the above mentioned additions (minimal medium). Cells were grown in T25 Primaria™ tissue culture flasks (BD Biosciences Benelux N.V, The Netherlands) at 37°C under 5% CO_2_ humidified atmosphere.

### Hormone stimulations and expression microarray analysis

Cells were seeded in their respective selection medium to reach ∼50% confluency and allowed to attach overnight. The next day, medium was replaced with 2% DCC-FCS in minimal medium and cells were starved for 48 h, to bring AR activity to basal levels before the hormone stimulations. Subsequently, cells were stimulated with either vehicle, 1 nM R1881 or 1 µM OH-flutamide for 4, 8 or 16 h. After stimulations, cells were rinsed twice with PBS and stored at −20°C until RNA isolation. Total RNA was isolated with RNAzol B reagent (Campro Scientific, Veenendaal, The Netherlands) and further purified through RNeasy columns (Qiagen) with on-column DNA digestion, according to the manufacturer's protocol. RNA quality was checked on 1% agarose gel.

Cy3- or Cy5-labelled RNA probes were produced by incorporating amino-allyl UTP during RNA amplification, followed by coupling to N-hydroxysuccinimide modified dye. Briefly, 3 µg RNA was used for a T7-based linear mRNA amplification protocol, described previously [9]. Amino-allyl UTP, plus equal amount of unmodified rUTP, was incorporated into aRNA with T7 Megascript Kit (all from Ambion), according to manufacturer's protocol. Amplified RNA was purified and concentrated using Microcon-YM 30 columns (Amicon®) to rinse three times with 300 µl RNAse-free water. Finally, 2 µg aminoallyl-modified RNA, in a maximum of 3.33 µl of RNAse-free water, was incubated with 1.66 µl sodium bicarbonate buffer (0.3 M, pH 9) and 5 µl Cy3- or Cy5-dye (CyScribe Post-Labeling Kit, Amersham, NJ, USA), for 1 h in the dark at room temperature. Reaction was stopped with 5 µl 4 M hydroxylamine HCl (Sigma), contra-labelled probes were combined and purified/concentrated using Microcon-YM 30 columns. Probe was collected in 5–15 µl final volume and resuspended in 80 µl Ambion hybridization buffer number 1.

For the microarray we used double-dye oligoarrays representing about 15,000 human genes, on which labelled hormone-stimulated RNA was cohybridized with its contra-labelled time-matched vehicle (ethanol) control. Two microarrays were performed per condition: in one experiment the stimulated samples were labeled with Cy3 and the unstimulated reference with Cy5, in the other experiment in vice-versa (dye-swap); this was done to exclude dye-preferential binding to oligonucleotides on the microarray. In addition, two independent cell passages were used for each of these experiments, to account for the biological variability.

The oligoarrays used in this study were produced at the Erasmus Center for Biomics. Briefly, a human 18,584 oligonucleotides library (Compugen, Sigma-Genosys) was spotted on aminosilane slides using a Virtek Chipwriter Professional arrayer (Virtek Vision International, Waterloo, Canada). Control spots included landmarks, spotting buffer, alien oligonucleotides (SpotReport Alien Oligo Array, La Jolla, Stratagene), poly d[A]40–60, salmon sperm DNA, and human COT-1 DNA. Before the hybridization, microarray slides were prehybridized in 5x SSC, 0.05% SDS, 4% BSA solution for 30 min at 45°C, washed twice with RNAse-free water for 2 min, rinsed with isopropanol and spin-dried for 3 min at 1500 g. Microarray hybridizations were performed overnight at 45°C, with continuous agitation, in a HS4800 Hybridization Station (Tecan Benelux BV). Finally, the arrays were washed automatically in the Hybridization Station using: 2x SSC/0.05% SDS (at 45°C), 1x SSC and 0.2x SSC (at room temperature), and dried under a stream of N_2_, before scanning.

### Data extraction and analysis

Arrays were scanned in a ScanArray Express HT scanner (Perkin Elmer, Nederland BV) and spot intensities were quantified using Imagene software (Bio Discovery Inc, El Sequndo, CA, USA). To balance Cy3 and Cy5 spot intensities, Loewess normalization per subarray was performed using limma-package (http://bioinf.wehi.edu.au/limma/) from Bioconductor (http://www.bioconductor.org) [10], [11]. To scale between arrays, the global median intensity per array was set at 1000. Dye intensities below 200 were then thresholded at 200, to minimize noise and make fold-change on the low-intensity range more robust against outliers. Spots with intensities below the threshold (200) for both Cy3 and Cy5 channels, in more than 50% (>3/6) of the arrays for each time-course, were excluded from the analysis. Sample to vehicle-control ratios were then calculated and 2log transformed. Spots that showed opposite effects for the dye-swap/biological replicates were excluded from further analysis; effects were called opposite if the mean 2log ratio for the three time-points tested were ≥0,5 for one dye and below ≤-0,5 for the dye-swap. Following normalization and all the above-mentioned quality controls, the 2log intensity ratios from both replicates were averaged for each time point. This data was stored in SRS7 (Sequence Retrieval System version 7, Lion Bioscience AG, Heidenberg, Germany) [12], which was also used for the comparisons with other previously published/publicly available databases [13], [14], [15], [16], [17], [18], [19], [20], [21], [22], [23], [24].

Hierarchical clustering and data visualization was performed using Cluster and TreeView programs (Eisen Labs: http://rana.lbl.gov), respectively. Significance Analysis of Microarrays (SAM; http://www-stat.stanford.edu/~tibs/SAM) was used to determine which genes were statistically different between stimulated samples and non-stimulated references. Gene ontology clustering was performed using Database for Annotation, Visualization and Integrated Discovery (DAVID: http://david.abcc.ncifcrf.gov) [25], [26]. The pathway and functional analyses were generated through the use of Ingenuity Pathways Analysis (Ingenuity® Systems, www.ingenuity.com).

All the microarray data is MIAMI compliant and has been deposited in the Gene Expression Omnibus repository (http://www.ncbi.nlm.nih.gov/geo/), under the GEO accession number GSE22914.

### cDNA synthesis and RT-PCR analysis

Total RNA was isolated as described above and cDNA was synthesized using MMLV-reverse transcriptase kit and Oligo(dT)_12–18_ primer (Invitrogen), according to manufacturer's protocol. cDNA samples were stored at −20°C. For the validation of the microarray results, quantitative real-time PCR analysis was performed using an ABI Prism 7700 Sequence Detection System (Applied Biosystems, Foster City, CA). KLK2, PART1, TPD52, FKBP5, GPR88, STEAP1, TRIB1 and ID3 were quantified with ABsolute QPCR SYBR Green ROX Mix (Thermo Scientific) and 330 nM of each primer, according to the manufacturer's protocol. Primers were designed using the computer program Oligo Primer Analysis Software version 6.22 (Molecular Biology Insights Inc, USA). Gene specificity was checked by BLAST and, whenever possible, intron-spanning primers were chosen to avoid amplification of contaminating DNA. Primer sequences are described in Table 2. TMPRSS2, PSA and GAPDH were quantified by TaqMan real-time PCR analysis, using ABsolute QPCR ROX Mix (Thermo Scientific). TMPRSS2 (assay ID Hs00237175_m1) and GAPDH (assay ID Hs99999905_m1) kits were purchased from Applied Biosystems and run following the manufacturer's instructions. PSA was quantified as described previously [8]. For each gene, a standard curve was constructed from serial dilutions of a reverse-transcribed PC346 RNA pool, which was then used to determine the quantity of target message from the threshold cycle (Ct) value. The GAPDH housekeeping gene was used as endogenous control.

**Table 2:** Primer sequences used in the quantitative RT-PCR analysis.

Gene	Forward primer	Reverse primer
KLK2	AGATGAAGACTCCAGCCAT	GATACCTTGAAGCACACCA
PART1	GAGCCAGCCAATCACTT	AGCAGCACTCAGGCGT
TPD52	TTTCAATGTGTTGGAAACTGTAA	TAGAATACCTTGGCCTCTATGC
GPR88	CCAAGGCGTCTCTTTAAGT	ATGGCAACTCATACTGGTG
FKBP5	GAATACACCAAAGCTGTTGA	CTCTTCCTTGGCATCCT
STEAP1	AGAAGATGCCTGGATTGA	CTTCTTCCTCAAGCATGG
ID3	GGAGCTTTTGCCACTGACTC	GCTCCTGAGCACCAGGTTTA
TRIB1	ATGGGACTTTGAGAAGAGG	GCCATCTCACTGTTCACAT

For the quantitative PCR analysis of the human tissue panel, normal and tumor samples from patients were obtained from the frozen tissue bank of the Erasmus Medical Center (Rotterdam, the Netherlands). Additional information about these specimens was provided previously [27]. TaqMan real-time PCR analysis was performed in an ABI Prism 7700 Sequence Detection System (Applied Biosystems, Foster City, CA), using AmpliTaq Gold DNA polymerase (Applied Biosystems), according to manufacturer's specifications. Validated primers and probes from TaqMan Gene Expression Assays (Applied Biosystems) were used for quantification of ACSL3 (Hs01071247_m1), MCCC2 (Hs00223257_m1), ENDOD1 (Hs00826684_m1) and GAPDH (Hs99999905_m1), according to the PCR settings provided by Applied Biosystems. PBGD was quantified using 330 nM of primers forward: 5′-CAT GTC TGG TAA CGG CAA TG-3′ and reverse: 5′-GTA CGA GGC TTT CAA TGT TG-3′ primers, in Power SybrGreen PCR Master mix (Applied Biosystems), according to thermocycling protocol recommended by the manufacturer. Transcript quantities for each sample were normalized against the average of two endogenous references and relative to a calibrator. The two housekeeping genes used as endogenous references were PBGD and GAPDH; a mixture of cDNAs from prostate carcinoma xenografts was used as the calibrator. Graphs and statistics were performed with GraphPad Prism (version 3.0). P-values <0.05 were considered significant.

## Results

### Gene expression pattern of PC346 cells treated with R1881 and hydroxyflutamide

To characterize the expression profile of androgen receptor target genes in prostate cancer cells, we used expression microarray analysis on the PC346 cell line panel incubated with the androgen analogue R1881 or the antiandrogen OH-flutamide. The PC346 model system is composed of four cell lines: the androgen-sensitive PC346C and three hormonal therapy-resistant sublines, derived from the parental PC346C by long-term androgen ablation (PC346DCC), supplemented with the antiandrogen OH-flutamide (PC346Flu1 and PC346Flu2). All these sublines exhibit different properties with respect to AR status and responsiveness (summarized in Table 1) [8].

For the expression analysis we stimulated the cells with 1 nM R1881 or 1 µM OH-flutamide for 4, 8 or 16 h and cohybridized the labeled RNA with its time-matched vehicle (ethanol) control. Two microarrays were performed per condition, using two independent cell passages in dye-swap, to account for the biological variability and potential dye-preferential effects. Early time-points were chosen in order to enrich for primary AR targets, and minimize indirect secondary targets.

The two replicates per time-point were averaged and a total of 107 differentially-expressed transcripts were selected to constitute the AR pathway signature: 74 up-regulated and 33 down-regulated by R1881 and/or OH-flutamide (Tables 3, 4, 5, 6). Spots were considered to be differentially-expressed if the absolute 2log ratio ≥0.5 (ratio ≥1.42 or ≤0.71) for all three time-points, for at least one cell type. Significance Analysis of Microarrays (SAM) was used to determine which genes were statistically different between stimulated samples and non-stimulated references. In the experimental design, we chose to perform the hormonal stimulations at 3 different time points so that transcripts with a faster or slower response would not be missed. However, the time effect was negligible: most androgen-regulated transcripts were differentially expressed at all time three time points and for the statistical analysis we decided to pool all the 3 time points per condition. In total, there were 253 SAM significant genes, with a false discovery rate (FDR) set at 0.05 (Tables S1, S2, S3, S4). From our 107 signature transcripts, considered differentially-expressed according to the above-mentioned selection criteria, 77 were statistically significant by SAM. Indeed, the expression of the remaining 30 (28%) transcripts of our AR-target signature varied across the 3 time-points, so that these did not reach statistical significance in the pooled SAM analysis. This variation cannot be explained by a apparently predominant kinetic pattern, nor can it be attributed to the 4 h time point in particular. Since temporal regulation was observed for such few transcripts, no analysis was performed on the dynamics of gene-expression variation across time. The expression ratios presented in the tables and Figure 4 are from the average of all three time-points per condition. Finally, the fact that a considerable number of SAM significant transcripts were not included in our AR-regulated signature was due to our choice to set the 2log ratio threshold at 0.5.

**Table 3:** List of genes up-regulated by R1881.

GenBank ID	HUGO_Symbol	Cytoband	Cell line	2log ratio	SAM q-value
NM_018674	ACCN4	2q35	PC346C	0.7	0.000
NM_004457	ACSL3	2q34-q35	PC346Flu1	2.0	0.000
NM_014109	ATAD2	8q24.13	PC346Flu1	0.8	0.000
AK027213	BBS10	12q21.2	PC346Flu1	0.8	0.000
NM_020235	BBX	3q13.1	PC346Flu1	0.8	0.007
AK024850	C2orf31	2q34	PC346Flu1	1.3	0.000
NM_006079	CITED2	6q23.3	PC346Flu1	1.0	0.000
AK026498	CYP2U1	4q25	PC346Flu1	1.5	0.000
NM_012062	DNM1L	12p11.21	PC346Flu1	1.4	0.000
NM_018456	EAF2	3q13.33	PC346Flu1	2.3	0.000
AK026517	EHF	11p12	PC346Flu1	0.6	0.000
AK022827	EIF2C3	1p34.3	PC346C / PC346Flu1	0.6 / 1.0	0.056 / 0.000
NM_012081	ELL2	5q15	PC346Flu1 / PC346Flu2	1.8 / 1.0	0.000 / 1.093
AF111849	ELOVL5	6p21.1-p12.1	PC346Flu1	1.5	0.000
AB020637	ENDOD1	11q21	PC346Flu1 / PC346Flu2	1.3 / 1.1	0.000 / 1.093
NM_019018	FAM105A	5p15.2	PC346Flu1	1.0	0.000
AK024648	FAM107B	10p13	PC346Flu1	0.8	0.007
AL137343	FAM84A	2p24.3	PC346Flu1	1.1	0.000
NM_004117	FKBP5	6p21.3-21.2	PC346C / PC346Flu1 / PC346Flu2	1.9 / 4.2 / 1.7	0.000 / 0.000 / 1.093
AK024715	FLJ21062 *	7q21.13	PC346Flu1	1.4	0.000
NM_020474	GALNT1	18q12.1	PC346Flu1	0.8	0.000
NM_005271	GLUD1	10q23.3	PC346Flu1	1.1	0.000
NM_002069	GNAI1	7q21	PC346Flu1	0.9	0.000
AB042410	GPR88	1p21.3	PC346C / PC346Flu1 / PC346Flu2	1.4 / 3.0 / 2.6	0.000 / 0.000 / 0.125
NM_001530	HIF1A	14q21-q24	PC346Flu1	1.2	0.000
NM_003543	HIST1H4H	6p21.3	PC346Flu1	1.7	0.000
M60721	HLX	1q41-q42.1	PC346Flu1 / PC346Flu2	0.7 / 0.6	0.000 / 1.093
NM_014642	IQCB1	3q13.33	PC346Flu1	0.8	0.139
NM_002241	KCNJ10	1q22-q23	PC346C	0.7	0.056
AL137384	KIAA1109	4q27	PC346C	0.6	0.027
NM_001206	KLF9	9q13	PC346Flu1	0.8	0.000
AF188747	KLK2	19q13.41	PC346C / PC346Flu1 / PC346Flu2	0.8 / 1.1 / 1.0	0.000 / 0.000 / 1.093
AK026375	LOC93622 *	4p16.1	PC346Flu1	1.0	0.000
NM_005461	MAFB	20q11.2-q13.1	PC346Flu1	1.1	0.000
NM_003010	MAP2K4	17p11.2	PC346Flu1	0.8	0.000
AB050049	MCCC2	5q12-q13	PC346Flu1	1.1	0.000
AK021627	MORC4	Xq22.3	PC346Flu1	1.2	0.000
AF142409	MS4A6A	11q12.1	PC346Flu1	0.8	0.450
NM_005956	MTHFD1	14q24	PC346C	0.6	0.000
NM_016498	MTP18 *	22q	PC346C	0.6	0.000
NM_000662	NAT1	8p23.1-p21.3	PC346Flu1	1.7	0.000
AF039944	NDRG1	8q24.3	PC346Flu1	1.6	0.000
NM_006096	NDRG1	8q24.3	PC346Flu1	2.6	0.000
AK026383	NDRG1	8q24.3	PC346Flu1	2.1	0.000
NM_005596	NFIB	9p24.1	PC346Flu1	0.8	0.077
NM_020529	NFKBIA	14q13	PC346C / PC346Flu1 / PC346Flu2	0.7 / 2.4 / 0.7	0.000 / 0.000 / 1.093
NM_016590	PART1 *	5q12.1	PC346Flu1	1.5	0.000
NM_006810	PDIA5	3q21.1	PC346C / PC346Flu1	0.5 / 1.4	0.000 / 0.000
NM_016166	PIAS1	15q	PC346Flu1	1.3	0.000
AF070670	PPM1A	14q23.1	PC346Flu1	0.7	0.000
NM_004156	PPP2CB	8p12-p11.2	PC346Flu1	0.9	0.000
NM_002923	RGS2	1q31	PC346Flu2	0.7	0.000
D16875	RHOB	2p24	PC346Flu1	1.6	0.000
AK001478	RHOU	1q42.11-q42.3	PC346C / PC346Flu1 / PC346Flu2	0.8 / 2.3 / 1.0	0.000 / 0.000 / 0.000
AB051826	RHOU	1q42.11-q42.3	PC346Flu1	2.5	0.000
NM_005627	SGK1	6q23	PC346Flu1	0.9	0.000
AB040914	SHROOM3	4q21.1	PC346Flu1	1.0	0.012
NM_004595	SMS	Xp22.1	PC346Flu1	1.1	0.000
NM_003082	SNAPC1	14q22	PC346Flu1	0.6	0.166
NM_003104	SORD *	15q15.3	PC346Flu1	1.2	0.000
NM_012449	STEAP1	7q21	PC346C / PC346Flu1 / PC346Flu2	0.8 / 1.7 / 1.0	0.000 / 0.000 / 1.093
AK026813	STEAP2	7q21	PC346Flu1 / PC346Flu2	1.1/ 0.7	0.000 / 0.000
NM_005656	TMPRSS2	21q22.3	PC346C	0.6	0.117
NM_005079	TPD52	8q21	PC346Flu1 / PC346Flu2	1.2 / 0.7	0.000 / 0.208
AF294628	TWSG1	18p11.3	PC346Flu1	1.0	0.000
NM_003115	UAP1	1q23.3	PC346Flu1	1.0	0.000
NM_003359	UGDH	4p15.1	PC346C / PC346Flu1	0.6 / 1.9	0.098 / 0.000
AK001647	USP40	2q37.1	PC346C	0.7	0.000
AB020676	WWC1	5q34	PC346Flu1	0.7	0.000
AK022814	ZBTB10	8q13-q21.1	PC346Flu1 / PC346Flu2	1.3 / 0.5	0.000 / 1.093
NM_006006	ZBTB16	11q23.1	PC346C / PC346Flu1 / PC346Flu2	0.9 / 1.6 / 1.5	0.000 / 0.000 / 1.093
AF025771	ZNF189	9q22-q31	PC346Flu1	1.5	0.000
AL157445			PC346Flu1	1.0	0.007
D17210			PC346Flu1	1.0	0.000

*no approved HUGO symbol exist for this entry. If present, UNIGENE symbol is given in alternative.

The androgen-sensitive PC346C subline responded to the R1881 stimulation with increased expression of 18 genes, while 2 were down-regulated. Among these are some well-known AR regulated genes, such as KLK2, STEAP1, TMPRSS2 and FKBP5. The therapy-resistant sublines showed distinct responses to R1881 and OH-flutamide. PC346Flu1, which expresses 4-fold higher AR levels than the parental cell line, showed a “super-activation” of the AR pathway by R1881, not only in the magnitude of the gene expression but also in the number of regulated genes (20 androgen-regulated genes in the parental PC346C versus 91 in PC346Flu1). Conversely, the PC346DCC subline, which expresses residual levels of AR protein, showed no detectable changes in gene expression after the hormone treatments. Neither PC346C, PC346DCC nor PC346Flu1 showed significant alterations in the transcriptional profile in response to OH-flutamide. In contrast, PC346Flu2 cells, which express the T877A mutated AR, responded to both R1881 and this antiandrogen, although the response to the latter was weaker (14 genes up-regulated by R1881 versus 8 up-regulated by OH-flutamide; Tables 3 and 5, respectively).

### Validation of the microarray data

**Figure 1:**
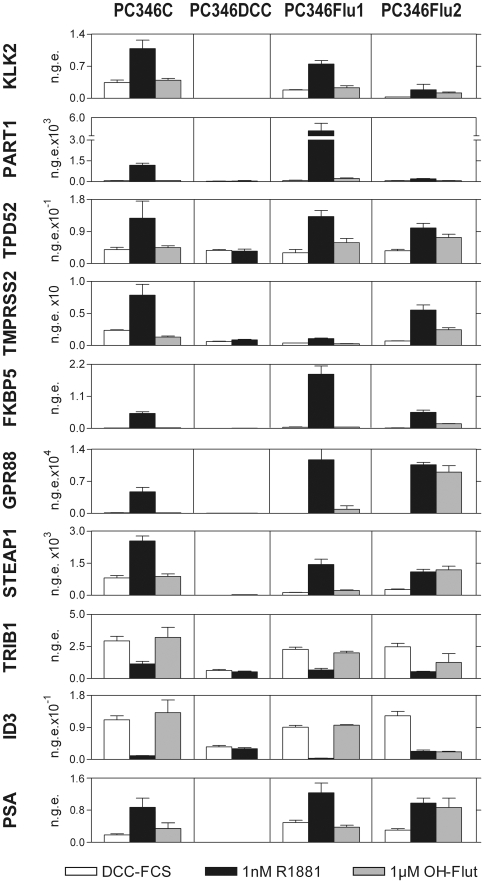
Validation of the microarray results. Quantitative RT-PCR analysis of a set of 10 androgen-regulated genes in PC346 cell lines. Cells were stimulated with 1 nM R1881 (R1881), 1 mM OH-flutamide (Flut) or vehicle control (DCC-FCS) for 16h. The graphs show the Mean (± SE) of the normalized gene expression (n.g.e.) relative to the housekeeping gene GAPDH, for two independent cell passages. ID3 and TRIB1 were androgen-repressed in the microarray assays, whereas the other genes were up-regulated by androgens.

The microarray data was validated by two approaches: an experimental approach using quantitative RT-PCR, and a bioinformatics approach linking our gene signature to a set of publicly available databases on androgen response. We selected 10 androgen-regulated genes to be further validated by quantitative RT-PCR: PSA, KLK2, PART1, TPD52, GPR88, FKBP5, TMPRSS2, STEAP1, ID3 and TRIB1. It is worth noting that our microarray analysis did not detect regulation of PSA expression in response to the hormonal treatments, but since this is a prominent AR target gene, it was included in the RT-PCR validation step. The quantitative PCR analysis confirmed the differential expression of all selected genes in the same direction predicted by the microarray analysis (Fig. 1). Furthermore, the RT-PCR also showed a stronger effect of the hormone-treatment on the PC346Flu1 cell line, in contrast to the almost absent induction of PC346DCC cells, when compared to the parental PC346C, for most genes analysed. As observed in the microarray assay, PC346Flu2 showed equivalent responses to R1881 and OH-flutamide for many regulated genes (Fig. 1, Tables 3, 4, 5, 6).

**Table 4:** List of genes down-regulated by R1881.

GenBank ID	HUGO_Symbol	Cytoband	Cell line	2log ratio	SAM q-value
NM_005688	ABCC5	3q27	PC346Flu1	−0.7	0.282
AK026288	ATHL1	11p15.5	PC346Flu1	−1.0	0.008
NM_012342	BAMBI	10p12.3-p11.2	PC346Flu1	−1.0	0.000
NM_001197	BIK	22q13.31	PC346Flu1	−0.8	0.112
AF075110	C14orf4	14q24.3	PC346Flu1	−1.3	0.000
NM_017766	CASZ1	1p36.22	PC346Flu1	−1.0	0.112
NM_001305	CLDN4	7q11.23	PC346Flu1	−0.6	0.282
AK024378	FAM131A	3q27.1	PC346Flu1	−0.7	0.052
NM_004480	FUT8	14q24.3	PC346Flu1	−1.0	0.316
NM_002165	ID1	20q11	PC346Flu1 / PC346Flu2	−0.8 / −1.1	0.018 / 1.087
X69111	ID3	1p36.13-p36.12	PC346Flu1	−1.3	0.000
NM_006769	LMO4	1p22.3	PC346Flu1	−0.8	0.000
NM_017572	MKNK2	19p13.3	PC346Flu1	−0.7	0.088
NM_005377	MYCL2	Xq22-q23	PC346C	−1.0	0.116
NM_006312	NCOR2	12q24	PC346Flu1	−0.6	0.052
U90907	PIK3R3	1p34.1	PC346Flu1	−1.0	0.008
AF113132	PSAT1	9q21.2	PC346Flu1	−0.8	0.000
NM_004577	PSPH	7p15.2-p15.1	PC346Flu1	−0.9	0.041
NM_015923	SLC3A2	11q13	PC346Flu1	−0.6	0.263
NM_003943	STBD1	4q24-q25	PC346Flu1	−0.8	0.022
NM_003714	STC2	5q35.1	PC346Flu1	−0.6	0.088
AK000401	TANC1	2q24.2	PC346Flu2	−0.7	1.087
AL133074	TP53INP1	8q22	PC346Flu1	−1.3	0.000
NM_003287	TPD52L1	6q22-q23	PC346Flu1	−0.7	0.402
AF205437	TRIB1	8q24.13	PC346Flu1	−1.6	0.000
U55055			PC346Flu2	−1.0	1.087
NM_018588			PC346C / PC346Flu1	−0.6 / −0.6	0.194 / 0.422
AK022971			PC346Flu1	−0.7	0.450
AK022971			PC346Flu2	−0.7	1.087

**Table 5:** List of genes up-regulated by hydroxyflutamide.

GenBank ID	HUGO_Symbol	Cytoband	Cell line	2log ratio	SAM q-value
AB020637	ENDOD1	11q21	PC346Flu2	0.6	0.158
NM_004117	FKBP5	6p21.3-21.2	PC346Flu2	0.7	0.226
AB042410	GPR88	1p21.3	PC346Flu2	1.9	0.000
AK025585	PARS2	1p32.2	PC346Flu2	0.8	0.296
NM_019091	PLEKHA3	2q31.2	PC346Flu2	0.7	0.158
NM_002923	RGS2	1q31	PC346Flu2	0.6	0.000
AK026813	STEAP2	7q21	PC346Flu2	0.6	0.118
D17099			PC346Flu2	0.9	0.926

**Table 6:** List of genes down-regulated by hydroxyflutamide.

GenBank ID	HUGO_Symbol	Cytoband	Cell line	2log ratio	SAM q-value
NM_014805	EPM2AIP1	3p22.1	PC346Flu2	−0.8	0.301
NM_006854	KDELR2	7p22.1	PC346C	−0.8	0.118
AB028451	NCOR1	17p11.2	PC346Flu2	−1.2	0.301
AB046842	PPP4R4	14q32.12-q32.13	PC346Flu2	-0.7	0.301
NM_001269	RCC1	1p36.1	PC346Flu2	−0.7	0.301
NM_000370	TTPA	8q13.1-q13.3	PC346Flu2	−0.9	0.301

**Figure 2:**
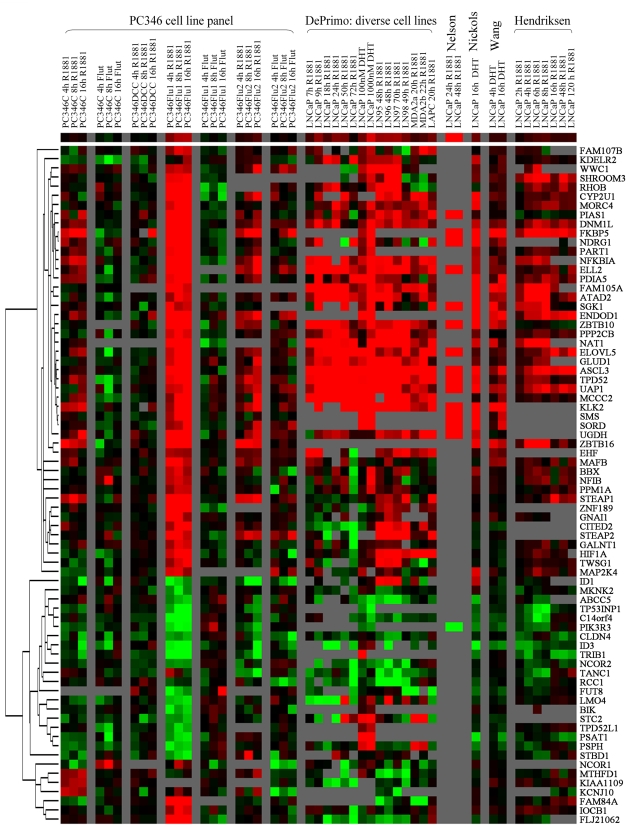
Expression profile of androgen-responsive genes in PC346 cells linked to publicly available databases on AR transcriptional regulation. On the left side, PC346C, PC346Flu1 and PC34Flu2 were exposed to 1 nM R1881 or 1 µM OH-flutamide for 4, 8 and 16h, whereas PC346DCC was stimulated with 1 nM R1881 only. On the right side, our gene signature was assessed in the databases from DePrimo *et al.*, Nelson *et al.*, Nickols *et al.*, Wang *et al.* and Hendriksen *et al.* (see Table 7 for database details). Heat-map is presented for the 2log expression ratio between hormone-treated samples and respective time-matched vehicle controls. Red and green colors represent induction and repression, respectively, whereas black indicates no regulation. Grey squares indicate missing data due to low expression, poor data quality or absence of probes for the respective transcript in the array platform used for the study.

In the past years, a series of studies have been published that analyzed gene expression in response to androgens stimulation in cell lines and xenografts (Table 7). Of the 107 transcripts in our signature, 73 were present in at least 3 of the 5 databases and were included for further analysis. More than 90% of the linked genes overlapped with previously reported androgen-regulated targets. Genes with the strongest inductions in our present work also showed consistently high inductions in multiple previous reports, suggesting that the products of these genes may play a basic role in the biological function of the prostate (Fig. 2). Using our unique cell line panel, we were able to identify novel androgen-responsive genes such as MAFB, KLF9, NFIB, STBD1, BIK and HLX.

**Table 7:** Description of the androgen-regulation and prostate cancer databases linked via SRS.

First Author (date)	Reference sample(s)	Query
DePrimo (2002) [13]	LNCaP, LN95, LN96, LN97, LN98, LAPC, MDA2a and MDA2b cell lines	1nM R1881, 10nM DHT, 100nM DHT and 1000nM DHT different time points from 7 to 72h
Hendriksen (2006) [14]	LNCaP	1nM R1881 time-course from 2h to 120h
Nelson (2002) [15]	LNCaP	24h and 48h 1nM R1881
Nickols (2007) [16]	LNCaP	16h 1nM DHT
Wang (2007) [17]	LNCaP	4h and 16h 100nM DHT
Best (2005) [18]	10 hormone-naive prostate cancers	10 hormone-refractory primary prostate tumors
Chandran (2007) [19]	64 primary prostate tumor samples	24 hormone-refractory metastatic samples from 4 patients
Lapointe (2004) [20]	41 benign prostate tissue adjacent to cancer	62 primary prostate tumor samples and 9 lymph node metastasis
Singh (2002) [21]	50 benign prostate tissue adjacent to cancer	52 primary prostate tumor samples: 8 recurrent and 13 non-recurrent (>4 years) after radical prostatectomy
Tamura (2007) [22]	10 hormone-naive prostate cancers	18 hormone-refractory primary and metastatic samples
Varambally (2005) [23]	4 benign prostate tissues	5 clinically localized prostate cancers and 5 metastatic samples
Yu (2007) [24]	60 benign prostate tissue adjacent to cancer and 23 disease free donor prostate tissue	62 primary prostate tumors and 24 metastatic samples from 4 patients

**Table 8:** Summary of significantly enriched Gene Ontology (GO) categories.

Annotation Cluster 1	Enrichment Score: 2.43	Count	P-value
	organic acid metabolic process	10	0.0025
	amino acid metabolic process	7	0.0046
Gene list: ACSL3, ELOVL5, PSPH, SMS, UGDH, GLUD1, MTHFD1, PPP2CB, MCCC2, PSAT1			

Annotation Cluster 2	Enrichment Score: 2.34	Count	P-value
	apoptosis	12	0.0019
	programmed cell death	12	0.0021
Gene list: DNM1L, PPP2CB, ELL2, BIK, RHOB, MTP18, TPD52L1, TP53INP1, SGK1, NFKBIA, ID3, ZBTB16			

Annotation Cluster 3	Enrichment Score: 2.27	Count	P-value
	developmental process	27	0.0088
	cell differentiation	19	0.0042
Gene list: FUT8, MAFB, MTP18, TPD52L1, CITED2, TTPA, SHROOM3, EHF, LMO4, NDRG1, TP53INP1, SGK1, BIK, RHOB, ZBTB16, UGDH, IQCB1, ID1, ID3, ELL2, DNM1L, HLX, KLF9, PPP2CB, TPD52, TWSG1, NFKBIA			

Annotation Cluster 4	Enrichment Score: 2.12	Count	P-value
	regulation of developmental process	7	0.002
	hemopoiesis	5	0.016
Gene list: MAFB, PPP2CB, TWSG1, NFKBIA, ZBTB16, SHROOM3, IQCB1			

Annotation Cluster 5	Enrichment Score: 1.69	Count	P-value
	negative regulation of metabolic process	7	0.023
	negative regulation of transcription	6	0.018
Gene list: PPP2CB, ZNF189, NCOR1, NCOR2, ID1, ID3, ZBTB16			

Annotation Cluster 6	Enrichment Score: 1.49	Count	P-value
	regulation of cellular process	31	0.014
	regulation of gene expression	23	0.017
Gene list: TP53INP1, SNAPC1, TPD52L1, ZBTB16, NFKBIA, ZBTB10, LMO4, NFIB, PIAS1, MKNK2, EHF, NCOR1, NCOR2, MAFB, KLF9, BIK, RHOB, BBX, ID1, ID3, HLX, ZNF18, TWSG1, IQCB1, PPM1A, RGS2, CITED2, PPP2CB, HIF1A, CASZ1, RCC1			

### Biologic processes coordinated by the AR pathway

The androgen-regulated signature genes were classified according to Gene Ontology (GO) Biological Processes using the Database for Annotation, Visualization and Integrated Discovery (DAVID: http://david.abcc.ncifcrf.gov) [25], [26].

**Figure 3:**
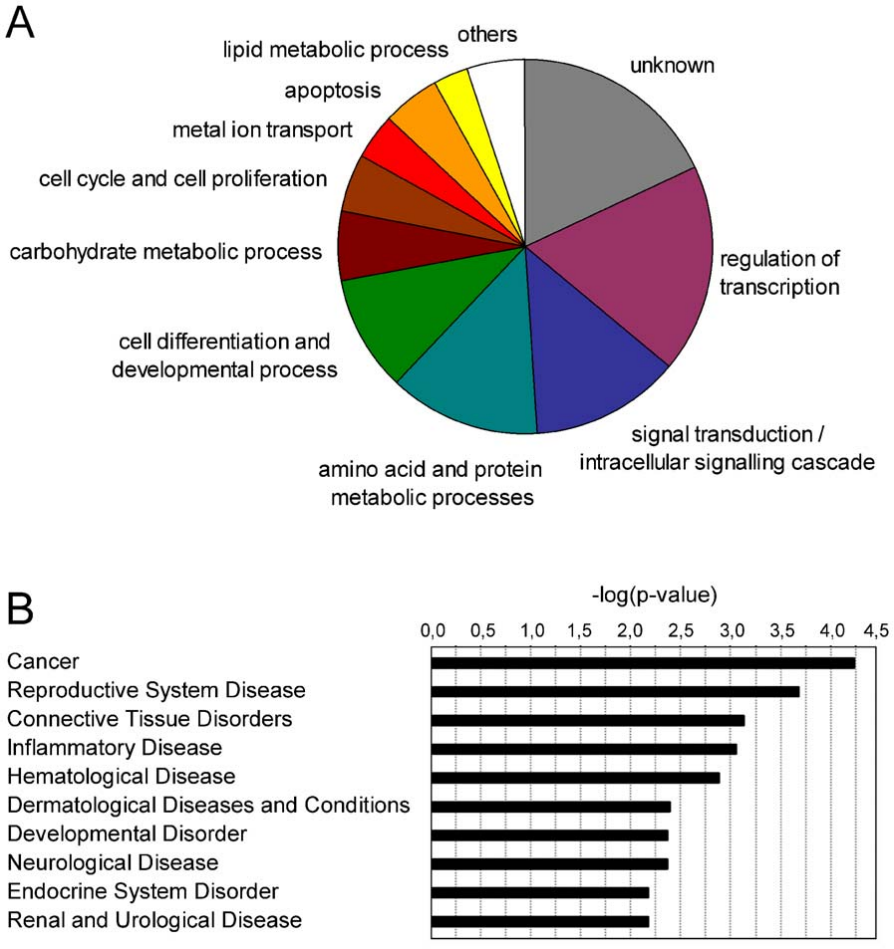
Biological processes regulated by the selected androgen-target genes. **(A)** Pie-graph representing genes categorized according to most prominent biological function. Gene ontology annotations were extracted using DAVID [25], [26]. **(B)** Involvement of the AR pathway genes in disease determined using Ingenuity Pathway Analysis (Ingenuity® Systems, www.ingenuity.com).

Consistent with the physiological roles of androgens, this approach revealed that the AR target genes selected in the present study operate in the regulation of transcription and intracellular signaling pathways, the metabolism of proteins, lipids and carbohydrates, and the regulation of cell proliferation and differentiation (Fig. 3A). The largest category includes genes encoding for transcription factors and transcription regulators, such as NFIB, KLF9, HIF1A, MAFB, EHF, NCOR1, NCOR2, PIAS1 and several zinc finger proteins (ZNF189, ZBTB10, ZBTB16 and CASZ1). This was followed by genes involved in intracellular signal transduction, including the G protein-coupled receptors pathway (GPR88, RGS2, GNAI1), small GTPases of the Ras family (RHOB, RHOU), mitogen-activated protein kinase cascade (MAP2K4, MKNK2, TRIB1) and other protein kinases/phosphatases (PPM1A, PPP2CB, PIK3R3, SGK1). Other AR responsive genes have an effect on cellular proliferation through regulation of cell cycle and apoptotic processes (e.g. RCC1, BBX, BIK, TP53INP1). Concomitant with the role of androgens on prostate development and maturation, another major cluster included genes involved in cellular differentiation, such as TPD52, TWSG1, NDRG1, ID1 and ID3. Finally, androgen induced the metabolism of proteins, carbohydrates and lipids that contribute to the production and secretion of prostatic fluid. Such R1881 target genes included MTHFD1, PSPH, PSAT1 and MCCC2, encoding enzymes in the metabolism of methionine, serine and leucine amino acids, respectively. Furthermore, up-regulation of the translation initiation factor EIF2C3 potentially promotes peptide synthesis. Moreover, genes participating in protein folding (PDIAS5, FKBP5), glycosylation (FUT8, GALNT1) and trafficking (DMN1L, KDELR2) were also regulated by R1881. Apart from proteins and amino acids, prostatic fluid is also rich in lipids, polyamines, sorbitol and several metal ions. Indeed, R1881 also stimulated expression of ACSL3 and ELOVL5, which participate in the elongation of fatty-acids, spermine synthase (SMS), part of the polyamine synthetic pathway, sorbitol dehydrogenase (SORD), secreted by the prostate into the seminal fluid, and the ion channels ACCN4 and KCNJ10.

To automate the functional classification, quantify the degree of enrichment of each cluster and select statistically significant functional categories we used the DAVID Functional Annotation Clustering tool. This tool identified 6 statistically significant Annotation Clusters, which associated with the metabolism of organic acids (lipids and amino acids), apoptosis, cell differentiation, developmental processes, regulation of transcription and regulation of cellular processes (Table 8).

The involvement of androgen-regulated genes in pathological conditions was further investigated by using the Ingenuity database (Ingenuity® Systems, www.ingenuity.com). The strongest associations were found for cancer, reproductive system, dermatological and cardiovascular diseases (Fig. 3B).

### The AR pathway in prostate cancer development and progression

**Figure 4:**
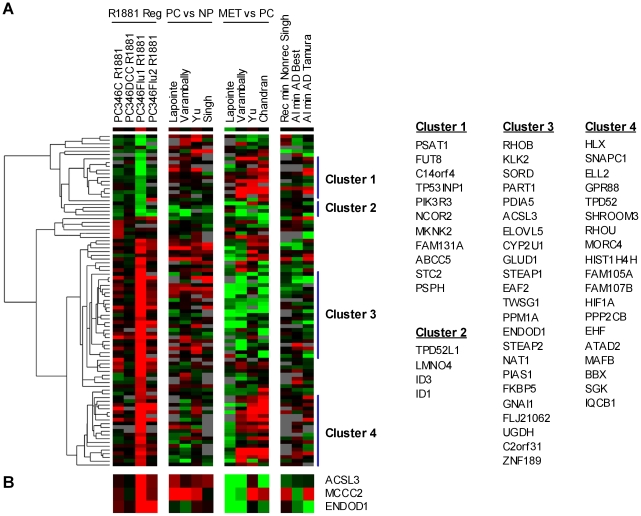
Expression of androgen-responsive genes in prostate cancer samples from patients. **(A)** Heat-map representation of publicly available expression data from human prostate cancer using the AR-regulated 107-genes signature (see Table 7 for database details). **(B)** Androgen-responsive genes selected for further quantitative PCR analysis. PC: prostate cancer; NP: normal prostate; Met: metastatic samples; AD: androgen-dependent; AI: hormonal therapy-resistant; (Non)Rec: (non)recurrent after radical prostatectomy. Color scheme as described for Figure 2.

To investigate how the AR pathway is modulated during the development and progression of prostate cancer we linked our androgen-regulated gene signature to seven independent prostate cancer microarray databases. These studies included specimens of “normal prostate” and prostate tumors from diverse disease stages, whose main characteristics are summarized in Table 7. A total of 89 hormone-responsive genes were present in at least 4 of the 7 databases, and were selected for further analysis. In figure 4, we show the hierarchical clustering of the R1881-responsive genes (first block of 4 columns), next to primary cancer versus normal prostate (second block), metastasis versus primary cancer (third block), and finally recurrent versus non-recurrent and hormonal therapy-resistant versus hormone-naïve disease (fourth block). The clustering analysis revealed four major gene groups: R1881-repressed and up-regulated during progression to metastatic disease (Cluster 1), R1881-repressed and down-regulated during progression (Cluster 2), R1881-induced and down-regulated during progression (Cluster 3), R1881-induced and up-regulated during progression (Cluster 4). About one third of the R1881-regulated genes was differentially-expressed between primary tumors and normal prostate in at least two databases. To this group contributed mainly R1881-induced genes that showed up-regulation in prostate cancer. These are genes that play a role in the production of prostatic fluid and in secretory function of the prostate, including SORD, ACSL3, ELOVL5, FKBP5, PDIA5, GLUD1 and UAP1. However, when comparing metastatic cancer to primary tumors, 23 of the R1881-induced genes were down-regulated (Fig. 4, Cluster 3), while 11 androgen-repressed genes were up-regulated (Fig. 4, Cluster 1). In total, these two clusters made up a considerable fraction (40%) of the androgen-responsive genes, and their expression pattern in metastasis suggests that the AR pathway is selectively down-regulated at this stage of the disease. In contrast, another group of R1881-stimulated genes showed increased expression in metastasis compared to primary tumors (Fig. 4, Cluster 4). This cluster is enriched for genes involved in survival/cellular proliferation (MAFB, ELL2, TPD52, EHF, HIF1A, HLX and SGK) and cell remodeling/adhesion (RHOU, SHROOM3, MORC4, TWSG1). Conversely, a group of R1881-repressed genes down-regulated in metastasis included genes involved in cellular differentiation and development (ID1, ID3, LMO4 and TPD52L1) (Fig. 4, Cluster 2). Finally, we assessed the activation state of the AR pathway in recurrent and in hormonal therapy-resistant disease. The collection of datasets in this category is limited to three non-concordant databases: Best *et al*. and Tamura *et al*. compared hormone-naïve with hormonal therapy-resistant samples, Singh *et al*. evaluated biochemical recurrence following radical prostatectomy. Therefore, the overlap between the three databases was modest. Nevertheless, the general trend is the same as for the progression of primary cancer to metastatic disease: genes down-regulated in metastasis tend to be down-regulated in recurrent versus non-recurrent and/or hormonal therapy-resistant versus homone-naïve disease, and vice-versa. These results suggest that the common mechanisms may govern the progression to different states of prostate cancer disease.

### AR target genes as markers for disease diagnosis and prognosis

The last objective of this study was to identify genes that could possibly be used as markers in the diagnosis of prostate cancer or in predicting the course of disease. We selected three R1881-regulated genes to be analyzed by quantitative PCR on normal prostate and prostate carcinoma samples obtained in our institute: ACSL3, MCCC2 and ENDOD1. The human prostate specimens obtained in our institute have been previously validated for marker research [14]. For this purpose, the well-known prostate cancer markers Hepsin and AMACR have been tested as positive controls [28], [29]. Both markers showed significantly higher expression in the prostate carcinoma than in the normal prostate samples, confirming that our panel is representative and suitable for the research of novel diagnostic/ prognostic markers [14]. The candidate genes were selected based on their strong androgen-induction, potential pathological function but, most importantly, on the fact that their expression was confirmed to be altered across multiple prostate cancer databases analyzed (Fig. 4B). In this sense, ACSL3 seems to be slightly up-regulated in primary prostate tumors and strongly repressed in metastatic cancer. Furthermore, fusion of the ACSL3 gene to the ETS family member ETV1 has recently been reported, making it an interesting gene for follow-up [30]. MCCC2 was strongly up-regulated in primary cancer, although its expression in metastasis and hormonal therapy-resistant disease varies in the different databases (Fig. 4B). Finally, ENDOD1 was one of the strongest R1881-induced genes in our microarray profile, and showed decreased expression in metastasis and therapy-resistant tumors, suggesting a possible role in disease progression (Fig. 4B).

**Figure 5:**
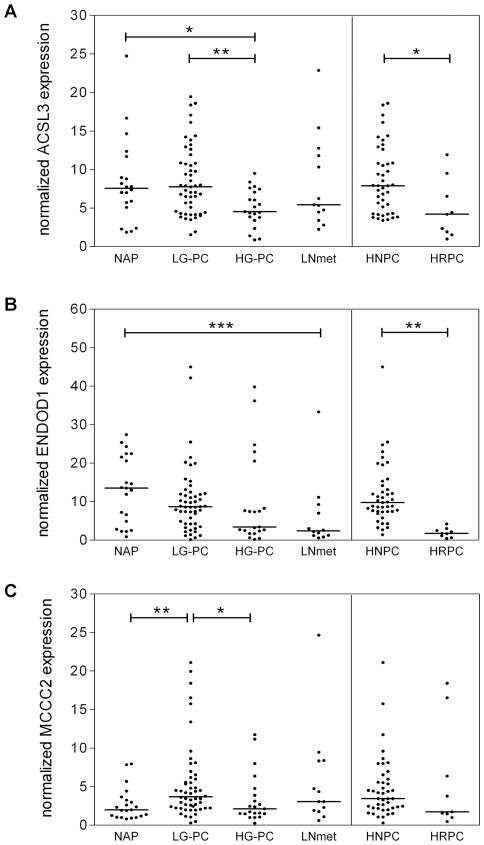
Quantitative RT-PCR analysis of three androgen-responsive genes ACSL3, ENDOD1 and MCCC2 in a distinct set of prostate samples. NAP: normal adjacent prostate; LG-PC: low-grade prostate cancer, including Gleason score from 5-7; HG-PC: high-grade prostate cancer, including Gleason score from 8-10; LNmet: lymph-node metastasis; HNPC: hormone-naïve prostate cancer (primary site); HRPC: hormonal therapy-resistant prostate cancer (primary site); (*) p-value ≤0.05 and (**) p-value ≤0.005 using Mann-Whitney two-tailed test; (***) p-value ≤0.05 with Post linear-trend test.

Quantitative PCR analysis included 21 samples of normal prostate tissue (adjacent to cancer), 73 primary prostate tumors and 13 lymph node metastasis. The primary tumors consist of 52 low-grade samples (Gleason 5–7), 21 samples from late-stage poorly-differentiated tumors (Gleason 8–10) and 9 hormonal therapy-resistant specimens, obtained from patients operated by radical prostatectomy or transurethral resection of the prostate (TURP). ACSL3 expression was significantly decreased during progression from low-grade to high-grade tumors (P = 0.005; Fig. 5A). ENDOD1 exhibited a stepwise down-regulation during disease progression (P<0.05 for Post linear-trend test), which is consistent with the results from the prostate cancer databases (Fig. 5B). Finally, MCCC2 was up-regulated in well-differentiated tumors (P<0.005), but its expression decreased during progression to high-grade cancer (P<0.05; Fig. 5C). This biphasic expression of MCCC2 during prostate cancer progression might explain the variation observed across the different databases mentioned above. Furthermore, expression of all three candidate genes was decreased during progression to therapy-resistant disease, although the trend for MCCC2 was not statistically significant.

## Discussion

In order to design better diagnostic and prognostic tools for prostate cancer and to develop more efficient therapies for late stage disease, it is essential to methodically understand the processes by which this disease develops and progresses. In this sense the AR pathway is of great interest for clinicians, researchers and pharmaceutical industry as it plays a crucial role in prostate malignancy. To investigate whether the AR pathway is functionally active in hormonal therapy-resistant prostate disease, we started by establishing the expression program of AR target genes in PC346 cell lines stimulated with R1881 or hydroxyflutamide.

R1881 stimulation of the androgen-sensitive PC346C subline resulted in differential expression of 20 genes, including the well-known AR target genes TMPRSS2, KLK2 and TPD52. Consistent with the expression of *wild-type* AR, OH-flutamide did not mediate transcription of AR-target genes in PC346C cells.

The castration-resistant PC346DCC subline, which expresses very low levels of the receptor, showed to be insensitive to R1881 stimulation. These results suggest that the AR pathway is not essential for the growth of PC346DCC cells. Alternative survival and growth pathways involved in the AR bypass in PC346DCC are under investigation and were recently published elsewhere [31].

Conversely, the cell line overexpressing the AR, PC346Flu1, showed a “super-activation” of the pathway, not only in the number of regulated genes but also in the strength of this regulation. This reveals two important aspects: (i) although these cells have been cultured in the absence of androgens for longer than two years, the AR is still functional and can be activated by the presence of its ligand; (ii) the AR seems to be hyper-sensitive, likely due to the high levels of the receptor, which may be sufficient to support cell growth under the hormone-depleted conditions. Interestingly, PC346Flu1 proliferation is inhibited by physiological concentrations of androgens, both *in vivo* and *in vitro*, suggesting that AR “super-activation” is unfavorable for cell growth, possibly by inducing cellular differentiation [8]. This is in line with a previous report, which showed that prostate epithelial cells tolerate a narrow-range of AR expression and activity, by undergoing apoptosis in the absence of AR expression and cell cycle arrest upon AR hyper-stimulation [32]. How AR overexpression can lead to receptor activation under androgen-depleted conditions is still not fully understood. Hypothetically, there are two possible mechanisms: increased sensitivity to residual androgen levels or constitutive ligand-independent activation. Some authors have proposed that increased AR levels not only sensitised the receptor to residual androgen concentrations but also conferred agonistic activity to AR antagonists [33], [34]. However, previous results showed that PC346Flu1 proliferation was optimal in the absence of androgens and was unaffected by flutamide supplementation [8]. The lack of agonistic activity of flutamide on PC346Flu1 cells was further confirmed in the present expression microarray analysis. Likewise, Konkontis et al. also failed to replicate the antagonist to agonist conversion in hormone-refractory LNCaP-104R cells, which express 15-fold more AR protein that respective androgen-sensitive parental LNCaP-104S cells [35], [36]. These results suggest that the proliferation of PC346Flu1 cells is not dependent on residual androgens, but is maintained by constitutive AR activation resistant to AR antagonists. This view is supported by findings from Dehm et al., which, by introducing disabling mutations in the ligand-binding domain, showed that ligand binding was not necessary for constitutive AR activation in C4-2 cells [37]. The authors also observed increased transactivation activity of the AR N-terminal domain in these cells, compared to parental LNCaP. Similar processes could be playing a role in constitutive AR activation in PC346Flu1 cells. In contrast, a recent report by Waltering et al., supports the hypothesis of increased sensitivity to residual androgen levels upon 2 to 4 times (LNCaP-ARmo) and 4 to 6 times (LNCaP-ARhi) LNCaP- AR overexpression [38]. Additionally, the authors also analysed the androgen response of these cell lines by expression microarrays. About 2/3 of the AR-regulated genes in our signature were also regulated upon DHT treatment of LNCaP-ARmo and/or LNCaP-ARhi. In particular, this included genes involved in secretory pathways, lipid and sugar metabolism (such as, UGDH, SORD, GLUD1, ELOVL5, ASCL3, UAP1), but also genes implicated in tumor progression and metastasis with functions in cell survival, proliferation and adhesion (EHF, ELL2, TPD52, MAFB, SGK). All together, AR overexpression may lead to different mechanisms of activation, depending on the background of the cells, the type or the duration of the androgen-depletion treatment.

In PC346Flu2 subline, carrying a mutated receptor, transcription of AR-target genes was regulated by both R1881 and OH-flutamide, although the stimulatory effect of the latter was weaker. This is in agreement with the agonistic action of OH-flutamide on the T877A mutated AR in promoting rather than inhibiting the growth of PC34Flu2 cells [8], [39].

In general, from these analyses we can conclude that the AR pathway is modified and still able to respond to stimuli, in the majority of therapy-resistant prostate cancer cells subjected to long-term androgen ablation. Furthermore, it is worth noting how the AR transcription patterns of the three therapy-resistant sublines reflected their respective AR modifications and growth characteristics. AR down-regulation correlated with deficient activation of AR-target genes; high-levels of AR resulted in more differentially-expressed genes and stronger regulation upon R1881 stimulation; finally, the T877A mutated AR responded to both R1881 and hydroxyflutamide.

To investigate the biologic processes coordinated by the AR target genes we used DAVID and Ingenuity tools to extract and cluster Gene Ontology Annotations. Consistent with the physiological roles of androgens in prostate development and maturation, the selected gene-signature is enriched for functions in transcription regulation, intracellular signal transduction, differentiation and regulation of cell proliferation and cell death. Further functions are associated with the metabolism of proteins, lipids and carbohydrates, which can be related to the production and secretion of prostatic fluid. Pathway analysis using Ingenuity showed strong association of the androgen-regulated genes to pathological conditions as cancer, reproductive system, dermatological and cardiovascular diseases (Fig. 3 and Table 8).

Next we evaluated the role of the AR pathway in prostate cancer development and how it is modulated during cancer progression by linking our androgen-regulated gene signature to seven previously published microarray databases on clinical tumor samples. Together, these databases comprise 178 “normal prostate” samples and 331 malignant specimens, including metastasis, recurrent tumors and hormonal therapy-resistant samples (Table 7). It is worth noting that the definition of “normal prostate” is not the same across the different studies. While most authors used benign tissue adjacent to the tumor as the “normal" reference, Yu *et al*. used normal prostatic epithelia from individuals without evidence of prostatic disease [24]. They showed that the expression profile of prostate cells was not only altered within the tumor itself, but alterations were also detected in apparently benign tissue around the borders of the tumor. This so-called field-effect has been reported in various other studies, and it is believed to be more evident the closer the distance to the tumor [28], [40], [41]. Disparity in the sampling of the “normal prostate” reference may certainly contribute to the variation seen between the diverse studies, together with differences in study design, microarray platforms, and most importantly, in the characteristics of the tumors included.

In summary, our AR-response profiling revealed that a considerable fraction of AR pathway genes were up-regulated in primary prostate cancer compared to normal prostate and down-regulated in metastasis. Further inspection of this gene cluster showed enrichment for genes involved in differentiation and secretory function of the prostate, functions which are redundant, if not detrimental for progressing cancer cells (Fig.4, Cluster 3). On the other hand, the cluster of androgen-regulated genes over-expressed in metastasis is enriched for genes involved in cell survival, proliferation, cytoskeletal remodelling and adhesion, all crucial functions in tumor progression and invasion (Fig. 4, Cluster 4).

It is generally accepted that the AR pathway accounts for the tumor growth in most prostate cancer patients even under hormonal ablation therapy. This hypothesis is supported by numerous reports that the AR itself is expressed in the majority of prostate cancers and often amplified in metastasis and therapy-resistant tumors [42], [43], [44], [45], [46]. Chen *et al*. have shown that AR overexpression is the most common modification following androgen ablation treatment, and is sufficient to confer hormonal therapy-refractory growth [33]. Furthermore, clinical tumor relapse is determined by PSA recurrence, which may give the impression that the AR pathway has become again fully functional. However, the results of our present study showed a selective down-regulation of AR target genes, questioning the over-simplistic view of the AR pathway as the driving force for prostate cancer growth and proliferation. In fact, the raise in serum PSA levels during relapse rather reflects the expansion of the tumor burden than increased AR activity in the tumor tissue self [47]. Indeed, Sterbis *et al*. reported that increased risk of biochemical recurrence was associated with low expression of tissue PSA mRNA [48]. Furthermore, the authors observed that serum PSA levels did not correlate with tissue mRNA expression, which was decreased in malignant compared to benign prostate epithelial cells [48]. By using distinct cell lines to establish the androgen-response signature and expanding the patient-derived database sets, our results corroborate previous observations from Hendriksen *et al*., which used the androgen-response expression profile from LNCaP cells to interrogate a set of prostate cancer xenografts and patient-derived samples [14]. Shortly thereafter, with distinct bioinformatics approaches, two other studies confirmed an attenuated androgen signaling signature in high-grade and metastatic prostate cancer, indicating that down-regulation of the AR pathway, although controversial, is likely to be a true phenomenon [49], [50].

The mechanisms for this selective modulation of the AR pathway during prostate cancer progression are yet undefined, but we speculate that it may be dictated by an imbalance in AR co-regulators and/or interactions with other signaling pathways. Indeed, alterations in several AR co-activators and co-repressors have been previously detected in prostate cancer and, in particular, in hormonal therapy-resistant disease [51], [52]. Furthermore, crosstalk between the AR and other growth factor pathways has been shown to activate AR signaling and selectively regulate a fraction of the AR transcriptional program, in response to IGF and EGF [53], [54].

**Figure 6:**
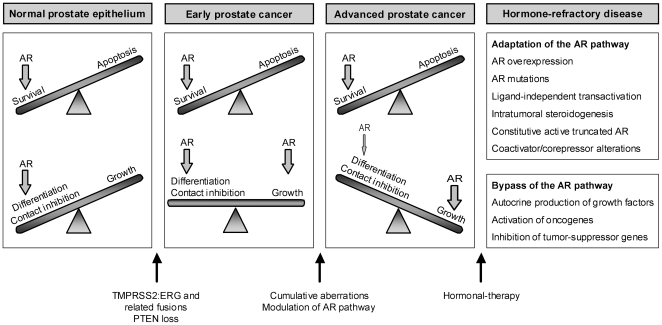
Proposed model for prostate cancer progression. In normal prostate epithelial cells the AR maintains the balance between survival, differentiation and proliferation. Cooperation between TMPRSS2:ERG (or related gene fusions) and PTEN loss (of one allele in early stages), is a potential mechanism suggested to drive the transition from prostatic intraepithelial neoplasia (PIN) to prostate adenocarcinoma. These aberrations are early events in prostate cancer development and are present in a large fraction of tumors. Initiation of prostate cancer is marked by a switch from androgen-dependent survival and differentiation to androgen-responsive proliferation. As cancer progresses, the balance is tilted towards tumor growth, while genes involved in prostate differentiation and secretory function are selectively repressed and genes promoting proliferation are up-regulated. In advanced disease, this mechanism eventually culminates in poorly-differentiated fast-growing tumors. Hormonal-therapy is offered to patients with advanced invasive disease, but the tumors will eventually become resistant to androgen ablation/blockade by either adapting or bypassing the AR pathway.

To accommodate these novel insights into our current knowledge of prostate cancer disease, we propose the following model for the development and progression of prostate tumors (Fig. 6): in the normal prostate the AR maintains prostate homeostasis and secretory functions through a delicate balance between cell survival and differentiation. A yet unknown trigger leads to a switch from androgen-dependent survival to androgen-stimulated cellular proliferation. Recent findings implicate gene fusions between androgen-regulated genes and ETS transcription factor family members in this process. The TMPRSS2-ERG fusion is the most frequent rearrangement, being detected in approximately 50% of the prostate tumors [55]. The androgen-responsive promotor region of the TMPRSS2 gene drives robust expression of ERG, an oncogene that is also frequently involved in chromosomal translocations in Ewing sarcoma, myeloid leukemia and cervical carcinoma [56], [57], [58]. Up to date, multiple other ETS family members and 5′ fusion partners have been identified in related rearrangements in prostate cancer [59], [60], [61], [62]. However, the biological role of ETS fusions in prostate cancer development is still controversial, since ERG and ETV1 by themselves, do not seem to be tumorigenic [60], [63]. Recent evidence suggests that ERG overexpression cooperates with PTEN loss in the progression from PIN to prostate adenocarcinoma [64], [65]. It is worth noting that PC346 cells do not carry the TMPRSS2-ERG or TMPRSS2-ETV1 fusions, nor show increased expression of these oncogenes (unpublished data). Therefore, it remains unclear which mechanism may drive androgen-sensitive growth of PC346C cells. Nevertheless, it is still possible that other less common fusions partners that we did not test yet may be involved. We hypothesize that at early stages, when tumors are well differentiated, expression of prostate-specific genes and genes involved in the production/secretion of prostatic fluid is maintained or even increased due to the growth of the epithelial cell compartment. As tumors progress and become more aggressive, genes involved in prostate differentiation and secretory function are selectively repressed, while genes promoting proliferation are up-regulated. This mechanism will eventually culminate in a fast-growing, poorly-differentiated late-stage disease (Fig. 6). Upon hormone therapy, cells may become resistant and resume growth by adaptations of the AR pathway and/or activation of alternative growth pathways (Fig. 6. Reviewed in [7]). Our cell line model represents two of these AR modifications: AR mutation (PC346Flu2) and AR overexpression (PC346Flu1), as well as AR pathway bypass through activation of oncogenes and tumor suppressor down-regulation (PC346DCC) [31]. However, AR knockdown experiments suggest that the AR pathway may remain vital for most therapy-refractory cells, as it induced apoptosis and inhibited growth of multiple castration-resistant cell lines and xenografts [66], [67], [68], [69], [70], [71].

In order to identify androgen-regulated genes that could possibly be used in the diagnosis/prognosis of prostate cancer, we selected from our 107-gene signature three androgen-regulated genes: MCCC2, ENDOD1 and ACSL3. Quantitative PCR analysis showed increased MCCC2 expression in early-stage, well-differentiated tumors, while ENDOD1 and ACSL3, were decreased in late-stage tumors and metastasis. In addition, we analyzed immunohistochemical data made available by the Human Protein Atlas, to check the expression of the cognate proteins in tumor samples. The Human Protein Atlas portal is a publicly available database with high-resolution images showing the spatial distribution of proteins in 46 different normal human tissues and 20 different cancer types, as well as 47 different human cell lines (www.proteinatlas.org). Data on ACSL3, MCCC2 and ENDOD1 protein expression is available for 3 normal tissue samples and 11 prostate tumors. Immunohistochemical staining showed moderate to strong cytoplasmic positivity in the glandular prostate cells for all the three potential markers. These results confirm that the candidate genes are indeed translated into proteins that can be detected in the tumor samples, which makes the development of potential diagnostic/prognostic assays feasible. To assess the prognostic value of these genes we compared primary prostate cancer that eventually developed distant metastasis after radical surgery with the non-recurrent tumors, but saw no significant differences (data not shown). Ultimately, the large inter-individual variation resulted in a poor separation between the diverse disease stages, even when the differences in expression were statistically significant. This limits the applicability of MCCC2, ENDOD1 or ACSL3 as independent diagnostic markers, by preventing the setting of an expression cutoff with both high specificity and sensitivity. However, the performance of these candidates may be improved in combination with other disease markers, such as PSA or ETS gene fusions, which has yet to be further evaluated in the diagnosis and prognosis of prostate cancer. Finally, the down-regulation of all three candidate genes in hormonal therapy-resistant compared to hormone-naïve disease is in agreement with an attenuation of the AR pathway, providing important clues on the mechanisms of prostate cancer progression.

In conclusion, the present study showed that castration-resistant PC346 cells maintained a transcriptional response to (anti)androgen stimulation, which was in accordance with the expressed AR modifications. By linking AR modifications with enhanced transcriptional function in therapy-resistant PCa cells, these results corroborate the hypothesis that the AR pathway is adapted and active in most cells refractory to hormonal therapy. The present study also showed that the AR pathway is selectively modulated during PCa progression, leading to repression of genes involved in cellular differentiation and up-regulation of anti-apoptotic and proliferation genes. The AR-responsive gene signature reported here provides a valuable tool to elucidate the mechanisms of this selective adaptation of the AR signalling, as well as to investigate novel disease markers for PCa progression and potential targets for therapy.

### Links

Bioconductor [http://www.bioconductor.org]

DAVID Database [http://david.abcc.ncifcrf.gov]

Eisen Lab [http://rama.lbl.gov]

Gene Ontology Omnibus (GEO)[ http://www.ncbi.nlm.nih.gov/geo/]

Ingenuity Pathway Analysis [http://www.ingenuity.com/]

Significance Analysis of Microarray Data [http://www-stat.stanford.edu/~tibs/SAM]

The Human Protein Atlas [http://www.proteinatlas.org/]

## Supporting Information

Table S1 List of SAM significant genes for PC346C cell line.
(XLS)

Table S2 List of SAM significant genes for PC346DCC cell line.
(XLS)

Table S3 List of SAM significant genes for PC346Flu1 cell line.
(XLS)

Table S4 List of SAM significant genes for PC346Flu2 cell line.
(XLS)
